# The Town-Level Prevalence of Chronic Lung Conditions and Death From COVID-19 Among Older Adults in Connecticut and Rhode Island

**DOI:** 10.5888/pcd19.210421

**Published:** 2022-06-30

**Authors:** Taylor Jansen, Chae Man Lee, Shu Xu, Nina M. Silverstein, Elizabeth Dugan

**Affiliations:** 1 Gerontology Department, University of Massachusetts Boston, Boston, Massachusetts

## Abstract

**Introduction:**

As of November 2021, older adults (aged ≥65 y) accounted for 81% of all deaths from COVID-19 in the US. Chronic lung diseases increase the risk for severe COVID-19 illness and death. The aim of this research was to examine the association between town-level rates of asthma and chronic obstructive pulmonary disease (COPD) and deaths from COVID-19 in 208 towns in Connecticut and Rhode Island.

**Methods:**

We conducted a multistep analysis to examine the association between town-level chronic lung conditions and death from COVID-19. Pairwise correlations were estimated and bivariate maps were created to assess the relationship between COVID-19 deaths per 100,000 people and 1) asthma prevalence and 2) COPD prevalence among adults aged 65 years or older. We used multiple linear regression models to examine whether chronic lung conditions and other town-level factors were associated with COVID-19 death rates in Connecticut and Rhode Island.

**Results:**

Initial bivariate correlation and mapping analyses suggested positive correlations between asthma and COPD prevalence and COVID-19 death rates. However, after controlling for town-level factors associated with chronic lung conditions and COVID-19 death rates, multiple linear regression models did not support an association, but town-level factors (African American race and Hispanic ethnicity, age ≥65 y, and low educational attainment) were significant predictors of COVID-19 death rates.

**Conclusion:**

We found significant associations between town-level factors and COVID-19, adding to the current understanding of the impact of social determinants of health on outcomes.

**Summary**

**What is already known on this topic?**

Chronic lung diseases, such as chronic obstructive pulmonary disease (COPD) and asthma, are among the top preexisting conditions identified by the Centers for Disease Control and Prevention that increase the risk for severe COVID-19 illness and death.

**What is added by this report?**

Town-level factors (African American race and Hispanic ethnicity, age ≥65 y, and low educational attainment) were significant predictors of COVID-19 death rates, adding to the current understanding of the impact of social determinants of health on outcomes.

**What are the implications for public health practice?**

Public health policy makers could focus on communities reporting high rates of chronic lung conditions among the older adult population to provide more testing and access to vaccinations.

## Introduction

As of November 2021, 81% of all US deaths caused by COVID-19 occurred among people aged 65 years or older (1). Chronic lung diseases, such as asthma and chronic obstructive pulmonary disease (COPD), are among the top preexisting conditions that increase the risk for severe COVID-19 illness and death (2). Patients with preexisting lung conditions, including asthma and COPD, are more likely than people without these conditions to be hospitalized and die of COVID-19 (3–6). Even after controlling for sex, race and ethnicity, body mass index, and 10 prevalent comorbidities, a study found that people with chronic lung conditions were significantly more likely to be admitted to intensive care units, require mechanical ventilation, and die of COVID-19 (3). Although the association between chronic lung conditions and COVID-19 has been established at the individual level (3–6), information about town-level associations is lacking. Thus, the objective of this study was to examine this town-level association measured by the prevalence of asthma and COPD among the population aged 65 years or older per town in Connecticut and Rhode Island.

We addressed the following research questions: 1) Are town-level prevalence rates of asthma and COPD among adults aged 65 years or older associated with town-level COVID-19 death rates? 2) After controlling for other confounding town-level factors associated with the study variables, is there an association between the town-level prevalence of chronic lung conditions among adults aged 65 years or older and COVID-19 death rates?

Connecticut and Rhode Island have higher rates of asthma and similar rates of COPD compared with average rates among the US population aged 65 years or older (7). Additionally, more than 90% of COVID-19 deaths in these 2 states have been among adults aged 60 years or older (8,9), making this study population an appropriate sample to investigate the association between rates of chronic lung conditions and COVID-19 death rates.

## Methods

We used data from several publicly available data sets: the Connecticut Department of Public Health and Office of Policy and Management (8), the Rhode Island Department of Public Health (9), the 2021 Connecticut Healthy Aging Data Report (HADR) (10), the 2020 Rhode Island HADR (11), and the US Census Bureau’s 2014–2018 American Community Survey (12). Institutional review board approval was not required because no individual-level data were used.

### Measures

**COVID-19 deaths**. The outcome of interest was the total number of COVID-19 deaths from March 2020 through October 7, 2021, for each reported geographic unit (towns and cities; hereinafter, towns) in Rhode Island (n = 39) and Connecticut (n = 169). We obtained these data from the Connecticut Open Data Portal (8) and the Rhode Island Department of Health (9). If a town reported fewer than 5 deaths but more than 0 deaths, the data were suppressed for reasons of confidentiality (9); data for 6 towns in Rhode Island were suppressed. Because each of these towns reported 1 to 4 deaths, we recoded them as having 1 death to represent towns with *any* reported deaths to distinguish them from towns with *no* deaths. We calculated population-adjusted COVID-19 death rates to account for differences in the population size of each town (N = 208 towns; total population range, 827–179,435).

**Chronic lung conditions**. Chronic disease measures reported in the HADR were obtained and estimated from the annual summaries of Medicare claims of beneficiaries aged 65 years or older in the 2016–2017 Medicare Beneficiary Summary Files (13). The HADR calculates chronic condition prevalence rates through clinical algorithms applied to individual Medicare fee-for-service claims (13,14). If a beneficiary 65 years or older in 2017 *ever* met the claims-based diagnostic criteria for asthma or COPD since 1999, they were considered *ever* diagnosed with the chronic condition. Criteria were having at least 1 Medicare claim for an inpatient, skilled nursing facility, or home health care or at least 2 hospital outpatient or Part B Medicare claims with appropriate diagnosis codes during a 1-year period. Thus, the prevalence rates of chronic conditions reported in the HADR may be slightly higher than the current prevalence rates among beneficiaries reported by other Centers for Medicare & Medicaid Services sources, because the HADR includes beneficiaries who were *ever* diagnosed since 1999 (14).

The asthma measure was coded as the prevalence rate of asthma among residents 65 years or older who *ever* met diagnostic criteria (14). COPD is a group of lung diseases that includes emphysema and chronic bronchitis, characterized by causing airflow blockage and breathing-related problems (15). The COPD measure was coded as the prevalence rate of COPD among residents 65 years or older who *ever* met diagnostic criteria (14).

**Covariates.** We obtained data on all covariates from the 2021 Connecticut HADR (10), the 2020 Rhode Island HADR (11), and the 2014–2018 American Community Survey (12). Previous research (16,17) identified factors associated with high COVID-19 death rates at the town and county levels. At the town level, increased household size and proportion of non–US-born residents and African Americans independently predicted increased COVID-19 death rates (16). County-level factors associated with increased COVID-19 infection and death were increases in household size and proportion of non–US-born residents, African American residents, low educational attainment, and the proportion of all commuters using public transportation (17). Thus, to account for potential confounding factors associated with town-level COVID-19 death rates, we included the following covariates: the average household size in each town, the percentage of the town population aged 65 years or older that was African American or Hispanic, the percentage of the town population aged 65 years or older that had less than a high school diploma, the percentage of all commuters using public transportation, and the proportion of non–US-born residents. In addition, poverty, obesity, smoking, and proportion of African Americans are community-level risk factors associated with asthma prevalence (18). Therefore, we added the following covariates: among residents aged 65 years or older, the percentage living in poverty, the percentage with obesity, and the percentage with tobacco use disorder. Finally, rurality was identified as a community-level factor associated with COPD (19); therefore, we included rural status as a covariate.

The HADRs include more than 190 indicators drawn from more than 20 data sources and reported at local levels (ie, zip codes, neighborhoods, towns, cities) (20). The HADR recodes and categorizes the race and ethnicity of the state population aged 65 years or older as White, African American, Asian, “other” race, and Hispanic/Latino. The HADR defines “other” race as persons reporting their race as Native Hawaiian or Other Pacific Islander, American Indian or Alaskan Native, other tribal entities, 2 or more races, or any other category not captured in previous categories (14). Educational attainment of the state population aged 65 years or older was coded into less than a high school diploma or GED (General Educational Development), high school diploma or some college, college degree, and graduate or professional degree. The percentage of the state’s population 65 years or older living in poverty is defined by the American Community Survey as the percentage of people aged 65 years or older reporting an annual household income at or below the federal poverty level. The average household size is defined by the American Community Survey as the average number of persons per household. We obtained data on the non–US-born population from the 2014–2018 American Community Survey (12); we recoded these data as the percentage per town. We obtained data on the rate of a town’s population that commutes to work and the percentage of all commuters using public transportation from the 2014–2018 American Community Survey (12). Town prevalence of obesity and tobacco use disorder among the population aged 65 years or older was obtained from the 2016–2017 Master Beneficiary Summary File (9) and coded as the prevalence rate among residents 65 years or older who ever met the diagnostic criteria (14). Towns were considered rural if they were inside a rural county as defined by the Office of Management and Budget (21).

### Analytic strategy

We combined data on all towns in both states for a total study sample of 208 towns. We conducted a multistep analysis to examine the association between the independent variables (town-level prevalence of asthma and COPD) and the dependent variable, COVID-19 death rates. First, we conducted pairwise correlations between the rate of COVID-19 deaths per 100,000 people and 1) asthma prevalence and 2) COPD prevalence. We calculated tertiles of asthma prevalence, COPD prevalence, and the population-adjusted COVID-19 death rates to represent low, medium, and high rates for asthma (low, 9.5%–12.8%; medium, 12.9%–14.71%; high, 14.72%–19.1%); for COPD (low, 11.1%–18.9%; medium, 19.0%–22.8%; high, 22.9%–33.7%); and COVID-19 death rate per 100,000 people (low, 0–101.8; medium, 103.2–237.8; high, 239.3–1,694.7). Next, we created 2 bivariate choropleth maps in ArcMap version 10.8.2 (Esri) to visualize the intersection of low, medium, and high levels of asthma, COPD, and COVID-19 death rates. Finally, to account for confounding factors influencing COVID-19 death rates and chronic lung conditions at the town level, we estimated a series of multiple linear regression models. We used the variance inflation factor (VIF) before estimating the linear regression models to test for multicollinearity among the study variables. The VIF for all study variables was less than 4.07, below the standard threshold of less than 10, suggesting that study variables were not significantly correlated with one another. In preliminary analyses (Models 1 and 2), we examined the main effects of the independent variables (the prevalence of asthma and the prevalence of COPD in the population aged ≥65 y) separately. Model 3 estimated the main effects of both chronic lung conditions together. Model 4 estimated the main effect of asthma alone on COVID-19 death rates controlling for factors found in previous literature to predict county-level COVID-19 (16,17) and asthma (18). Finally, Model 5 added in the main effect of COPD and rural status as a covariate to Model 4. We conducted all statistical analyses in Stata version 17 (StataCorp LLC). *P* values of ≤.05 were considered significant.

## Results

Across all towns in both states, the average rate of COVID-19 deaths per 100,000 people was 200.2 deaths per town (Table 1). The prevalence of asthma was 13.8%, and the prevalence of COPD was 21.2%. The average town population was 22,299 people and 3,662 people aged 65 years or older. Most of the overall sample was White (93.9%) and had attained some college (53.5%). Of the population aged 65 years or older, 23.5% had obesity and 10.1% had tobacco use disorder. In addition, 11.5% of the overall sample were not US born, 2.5% commuted to work using public transportation, and 6.0% of the population aged 65 years or older were living in poverty. The average household size was 2.5 people per home and did not differ across the 2 states. Finally, 19.7% of the overall sample lived in rural counties; 24.3% of the sample in Connecticut lived in rural counties, whereas Rhode Island had no rural counties (*P* < .001 for difference between 2 states).

**Table 1:** Descriptive Statistics for Study Sample and Bivariate Analyses, by State, Study of Town-Level Prevalence of Chronic Lung Conditions and Death From COVID-19 Among Older Adults in Connecticut and Rhode Island

Factor	Both states	Connecticut	Rhode Island	*t* Statistic
No. of communities	208	169	39	—
**Variable**				
Population aged ≥65 y with asthma, %	13.8	13.7	14.5	−2.29^a^
Population aged ≥65 y with COPD, %	21.2	20.9	22.2	−1.69
COVID-19 death rates per 100,000 people	200.2	196.9	214.4	−0.53
**Characteristic**				
Total population, no. (SD)	22,299 (26,832)	21,192 (25,564)	27,093 (31,679)	−1.24
Population aged ≥65 y, no. (SD)	3,662 (3,591)	3,477 (3,463)	4,467 (4,051)	1.56
Race and ethnicity of population aged ≥65 y, %				
African American	2.9	3.2	1.7	1.40
Asian	1.5	1.6	1.1	1.70
Hispanic/Latino	2.9	2.9	2.8	0.11
Other^b^	1.6	1.5	2.1	−1.17
White	93.9	93.6	95.2	−1.05
Education of population aged ≥65 y, %				
Less than high school diploma	12.3	11.4	16.3	−3.68^c^
High school diploma or some college	53.5	53.9	51.7	1.23
College degree	16.5	16.6	16.3	0.11
Graduate or professional degree	17.7	18.1	15.7	1.73
Population aged ≥65 y with obesity, %^d^	23.5	22.3	28.3	−7.66^c^
Population aged ≥65 y with tobacco use disorder, %^d^	10.1	10.1	10.4	−0.56
Population aged ≥65 y living at or below federal poverty level, %	6.0	5.3	8.7	−5.26^c^
Population not US born, %	11.5	9.0	22.4	−3.39^c^
Commute to work using public transportation	2.5	2.7	1.7	1.47
Average household size (SD)	2.5 (0.2)	2.5 (0.2)	2.5 (0.2)	1.92
Rural^e^	19.7	24.3	0	3.52^c^

Abbreviation: —, does not apply; COPD, chronic obstructive pulmonary disease.

a *P* < .05.

b Other race is defined in the Health Aging Data Report as Native Hawaiian or Other Pacific Islander, American Indian or Alaskan Native, and other tribal entries, ≥2 races, or any other race not represented in previous categories (10,11).

c *P* < .001.

d Prevalence of the condition is defined as being *ever* diagnosed with the condition in the Medicare Beneficiary Summary File since 1999 (13,14).

e Living in a town in a rural county as defined by the Office of Management and Budget (21).

Overall, the Connecticut sample was slightly more diverse, healthier, and wealthier than the Rhode Island sample. Connecticut had an average of 196.9 deaths per 100,000 people from COVID-19 per town (vs 214.4 in Rhode Island and 200.2 overall). In bivariate analyses, we found a significant difference in the prevalence of asthma among adults aged 65 years or older by state (Connecticut, 13.7%; Rhode Island, 14.5%) and in the overall sample (13.8%). Compared with Rhode Island, Connecticut had a lower rate of COPD (20.9%) and a higher rate of people aged 65 years or older with a graduate or professional degree (18.1%). The average town population aged 65 years or older in Connecticut (n = 3,477) was lower than in Rhode Island (n = 4,467) and overall (n = 3,662). We found a significant difference between states for rates of obesity and poverty; among residents aged 65 years or older, Connecticut had a lower prevalence of obesity (22.3%) and a lower rate of residents aged 65 years or older living in poverty (5.3%). Finally, Connecticut had a lower rate of non–US-born residents (9.0%).

Rhode Island had a higher prevalence than Connecticut of asthma (14.5%), COPD (22.2%), and obesity (28.3%) among people aged 65 years or older. Towns in Rhode Island had worse health, were poorer, older, and less educated than towns in Connecticut. In Rhode Island, of the population aged 65 years or older, 8.7% lived in poverty and 16.3% had less than a high school diploma. Finally, Rhode Island had more than twice as many non–US-born residents as Connecticut (22.4% vs 9.0%).

**Figure 1:**
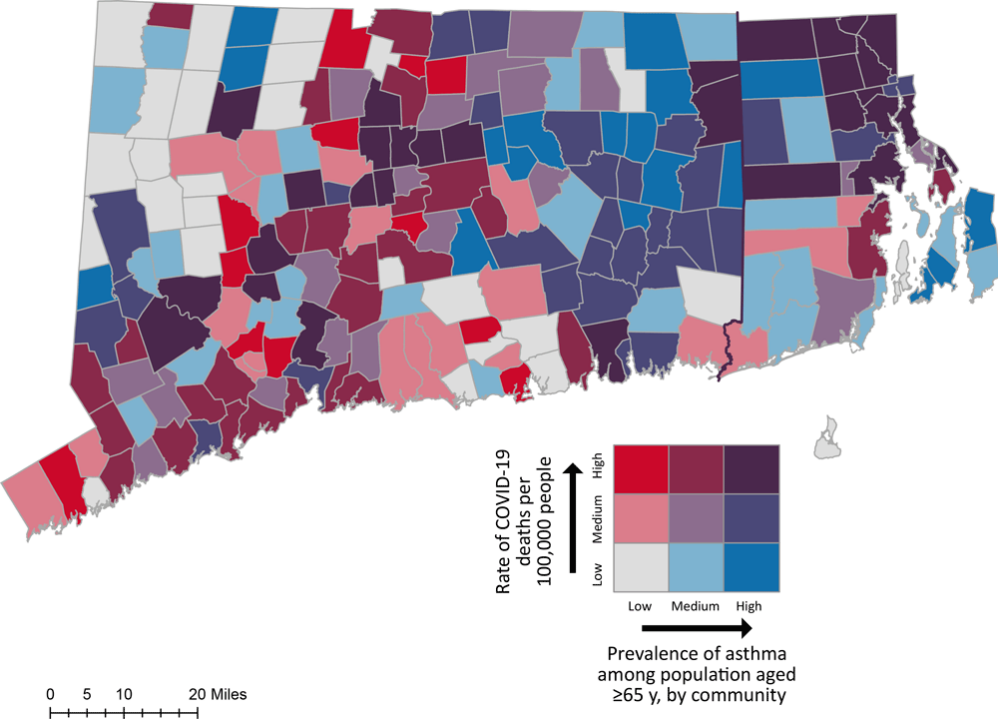
Town-level prevalence of asthma among population aged 65 years or older and COVID-19 death rates per 100,000 people, Connecticut and Rhode Island. Data sources: Connecticut Department of Public Health (8), Rhode Island Department of Public Health (9), HealthyAgingDataReports.org (10,11), CT DEEP GIS (22), and RIGIS (23).

**Figure 2:**
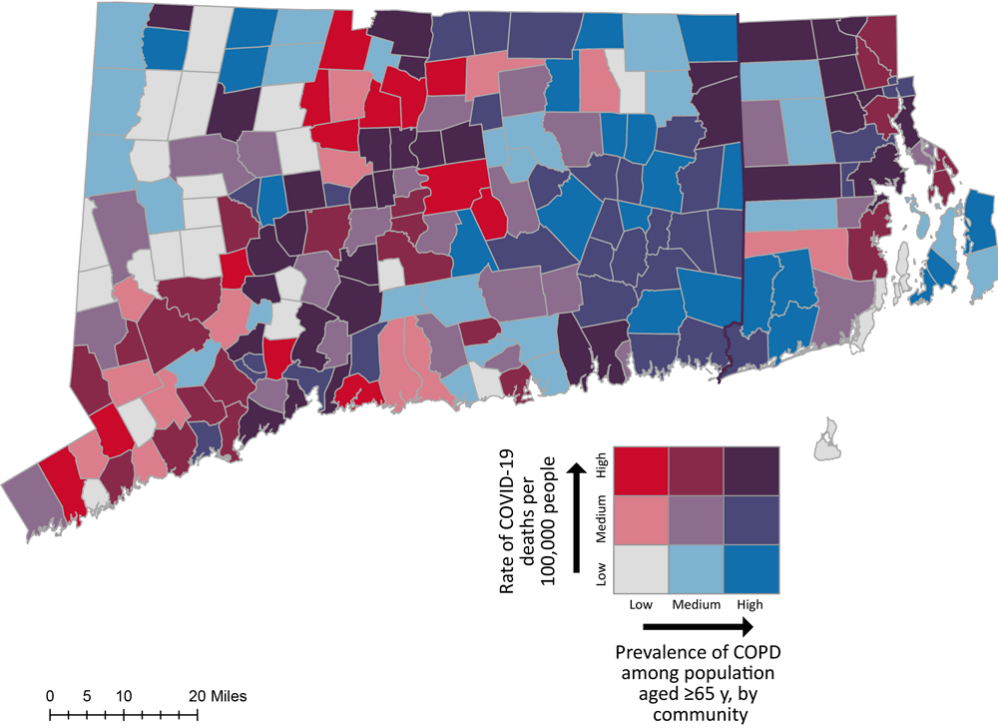
Town-level prevalence of COPD among population aged 65 years or older and COVID-19 death rates per 100,000 people, Connecticut and Rhode Island. Data sources: Connecticut Department of Public Health (8), Rhode Island Department of Public Health (9), HealthyAgingDataReports.org (10,11), CT DEEP GIS (22), and RIGIS (23).

We found significant correlations between town-level asthma prevalence among people aged 65 years or older and rate of COVID-19 deaths per 100,000 people (*r*[206] = 0.15; *P* = .03) and town-level COPD prevalence among residents aged 65 years or older and rate of COVID-19 deaths per 100,000 people (*r*[206] = 0.15; *P* = .03). Medium and high rates of chronic lung conditions and COVID-19 overlapped mainly in southwestern, central, and eastern Connecticut and in central and northern Rhode Island (Figure 1 and Figure 2).

In Model 1, the main effect of town-level prevalence of asthma among residents aged 65 years or older was significant, and the model positively predicted COVID-19 death rates (β = 0.15, SE, 619.02; 95% CI, 111.79–2,552.62; *P* = .03). Model 2 found a significant, positive association between town-level prevalence of COPD among residents aged 65 years or older and COVID-19 death rates per 100,000 people (β = 0.15; SE, 298.12; 95% CI, 48.78–1,224.3; *P* = .03). In Model 3, the significant effect disappeared (Table 2). Model 4 did not demonstrate a significant main effect for asthma (β = 0.02; SE, 860.31; 95% CI, −1,525.4 to 1,868.2; *P* = .84), but it did show a positive, significant effect in towns with a higher percentage of African American residents aged 65 years or older (β = 0.23; SE, 254.11; 95% CI, 191.86–1,194.26; *P* = .01) and residents aged 65 years or older without a high school diploma (β = 0.42; SE, 285.49; 95% CI, 454.55–1,580.72; *P* = .001). Model 5 also did not find a significant main effect between asthma (β = 0.04; SE, 893.12, 95% CI, 1,436.41–2,086.86; *P* = .72) or COPD (β = 0.02; SE, 568.27; 95% CI, −1,036.83 to 1,204.94; *P* = .99) and COVID-19 death rates, but multiple covariates had significant effects. Like Model 4, Model 5 showed that towns with a higher percentage of African American residents aged 65 years or older (β = 0.19; SE, 249.18; 95% CI, 83.8–1,066.8; *P* = .02) and residents aged 65 years or older without a high school diploma (β = 0.45; SE, 278.98; 95% CI, 529.06–1,629.6; *P* = .001), reported positive associations with COVID-19 death rates. In Model 5, which added COPD, a negative, significant effect was found for the Hispanic population aged 65 years or older (β = −0.23; SE, 407.43; 95% CI, −1,652.8 to −45.52; *P* = .04) and the population living in rural areas (β = −0.13; SE, 809.17; 95% CI, −184.92 to −55.95; *P* = <.001). These findings suggest that in our sample, as rurality and the percentage of residents who are Hispanic increased, COVID-19 death rates decreased.

**Table 2:** Results of Multiple Linear Regression for Association Between Town-Level Chronic Lung Conditions and Rate of COVID-19 Death Rates per 100,000 People, Connecticut and Rhode Island

Variable	Model 3,^a^ β (SE) [95% CI]	Model 4,^b^ β (SE) [95% CI]	*P* value	Model 5,^c^ β (SE) [95% CI]	*P* value
**Main effects**					
Prevalence of asthma among population aged ≥65 y^d^	0.09 (807.37) [−769.22 to 2,414.39]	0.02 (860.31) [−1,525.4 to 1,868.2]	.84	0.04 (893.12) [1,436.41 to 2,086.86]	.72
Prevalence of COPD among population aged ≥65 y^d^	0.09 (388.77) [−384.22 to 1,148.77]	—	—	0.02 (568.27) [−1,036.83 to 1,204.94]	.99
**Covariates**					
Percentage of residents aged ≥65 y that are African American	—	0.23 (254.11) [191.86 to 1,194.26]	.01	0.19 (249.18) [83.8 to 1,066.8]	.02
Percentage of population aged ≥65 y that are Hispanic	—	−0.19 (411.05) [−1,524.17 to 97.28]	—	−0.23 (407.43) [−1,652.8 to −45.52]	.04
Percentage of population aged ≥65 y without a high school diploma	—	0.42 (285.49) [454.55 to 1,580.72]	.001	0.45 (278.98) [529.06 to 1,629.6]	.001
Prevalence of obesity among population aged ≥65 y^d^	—	0.13 (376.24) [−246.84 to 1,237.28]	—	—	—
Prevalence of tobacco use disorder among population aged ≥65 y^d^	—	−0.17 (649.65) [−2,403.74 to 158.89]	—	−0.14 (809.17) [−2,464.49 to 727.64]	—
Percentage of population aged ≥65 y living at or below federal poverty level	—	−0.15 (523.69) [−1,795.81 to 269.96]	—	−0.15 (512.76) [−1,731.98 to 290.8]	—
Percentage of population not US born	—	−0.07 (0.56) [−1.63 to 0.57]	—	−0.09 (0.54) [−1.81 to 0.34]	—
Commute to work using public transportation	—	−0.09 (3.22) [−10.44 to 2.27]	—	−0.06 (3.17) [−9.24 to 3.26]	—
Average household size	—	−0.14 (68.97) [−264.59 to 7.48]	—	−0.17 (67.43) [−288.81 to −22.81]	.02
Rural^e^	—	—	—	−0.13 (809.17) [−184.92 to −55.95]	<.001

Abbreviations: —, does not apply; COPD, chronic obstructive pulmonary disease.

a Model 3: Constant = 5.44; adjusted *R* ^2^ = 0.02.

b Model 4: Constant = 435.81; adjusted *R* ^2^ = 0.11.

c Model 5: Constant = 537.6; adjusted *R* ^2^ = 0.16.

d Prevalence of the condition is defined as being *ever* diagnosed with the condition in the Medicare Beneficiary Summary File since 1999 (13,14).

e Living in a town in a rural county as defined by the Office of Management and Budget (21).

## Discussion

This study advanced the research by examining the association between town-level chronic lung conditions and mortality from COVID-19. First, pairwise correlations and bivariate mapping suggested an association between town-level chronic lung conditions (asthma, COPD) and death from COVID-19. The bivariate maps demonstrated multiple clusters in Connecticut and Rhode Island where the prevalence of asthma and COPD among residents aged 65 years or older overlapped with the rate of COVID-19 deaths at the town level. In a comparison of our bivariate maps and maps of variables from the 2021 Connecticut (10) and 2020 Rhode Island HADRs (11), the clusters in southwestern and central Connecticut in both maps are in an area of Connecticut with high proportions of people who are aged 65 years or older, African American, or Hispanic, or have low income and low educational attainment. Additionally, eastern Connecticut is largely rural and has high rates of asthma, COPD, obesity, tobacco use disorder, and older adults living in poverty. Northern Rhode Island also has high proportions of people aged 65 years or older, African American, or Hispanic, or who have low income, obesity, or low educational attainment (10,11). Although our bivariate maps demonstrated an association, a comparison of these maps with other maps of variables in the 2021 Connecticut and 2020 Rhode Island HADRs (10,11) showed that this association may be driven by other town-level factors.

In Model 4, although we found no significant effect for asthma prevalence predicting COVID-19 death rates among the population aged 65 years or older, the percentage of the population that was African American and aged 65 years or older and the population with less than a high school education had a positive, significant association with COVID-19 death rates. Furthermore, towns with a high proportion of older adults with low educational attainment were also more likely than towns without these characteristics to report higher rates of COVID-19 death rates. A previous study (17) also found that less than a high school education independently predicted at the county level a higher rate of COVID-19 deaths per 100,000 people across the US. Our study also found this effect at the town level among the population aged 65 years or older, underlining the robustness of this association. Thus, this finding suggests that towns with large populations of older adults with low levels of education are especially vulnerable to death from COVID-19.

The final model included both chronic lung conditions and rurality as the final covariate. Although we found no significant main effects, we found that the following covariates were significantly associated with COVID-19 death rates: percentage of population that was African American or Hispanic, aged 65 years or older, or had less than a high school education; average household size; and rurality. The positive, significant association between the African American population aged 65 years or older and mortality was slightly reduced, but the association became slightly stronger between the population that was aged 65 years or older with less than a high school education and COVID-19 death rates. Furthermore, we found a negative, significant effect between 1) the association between rurality and COVID-19 death rates and 2) the Hispanic population aged 65 years or older and mortality. These data suggest that rural towns and towns with large populations of Hispanic people aged 65 years or older had lower rates of COVID-19 deaths per 100,000 people than urban towns without large populations of Hispanic people aged 65 years or older. This finding is confounding because CDC reports that African American and Hispanic people are 1.9 and 2.1 times, respectively, more likely than White people to die of COVID-19 (24). Yet, the “Hispanic paradox” (25) indicates that although older Hispanic people are not healthier than older non-Hispanic people, they have lower rates of mortality and the highest life expectancy in the US (26). Future research could examine this relationship more closely. Finally, our study found that living in a rural county protected against COVID-19 death rates in Connecticut. However, previous research found that rurality is associated with higher rates of COVID-19 deaths at the county level (27). Our study used a population-adjusted indicator of COVID-19 death rates; only 2 counties in our sample were defined as rural, and both were in Connecticut. Population density may affect the transmission of highly contagious airborne viruses, and living in urban, densely populated areas may increase the everyday risk of COVID-19 exposure (28).

This study has several potential limitations. Although we found that more than 90% of COVID-19 deaths in Connecticut and Rhode Island were among the population aged 60 years or older, the measures of chronic conditions and population characteristics used represented the population aged 65 years or older, and the COVID-19 death rates data were reported for all ages in these states; thus, the age groups do not align exactly across data sources. Additionally, the research design was cross-sectional, so causation could not be determined. Furthermore, this study examined only asthma and COPD; perhaps the study of other common chronic lung conditions would show different results.

Our research fills a gap in the knowledge base of the association between town-level rates of asthma and COPD among older adults and COVID-19 death rates. Our analysis did not find that the prevalence rates of asthma and COPD among the population aged 65 years or older predicted COVID-19 death rates. Yet it did find significant associations between town-level factors and COVID-19 death rates, adding to the knowledge base indicating that large proportions of racial and ethnic minority groups and low educational attainment among the population aged 65 years or older are significant predictors of town rates of COVID-19 deaths.

Future research could examine how demographic indicators most relevant to older adults are associated with COVID-19 death rates at the town level and whether the indicators found for the overall population are the same for the older adult population. Finally, this research identified communities in Connecticut and Rhode Island at the highest risk for hospitalization and mortality from COVID-19. These communities would benefit from additional policy efforts that promote and provide COVID-19 testing and access to vaccination.
